# Isolation and characterization of 24 polymorphic microsatellite loci for the study of genetic population structure of the sheepshead *Archosargus probatocephalus* (Actinopterygii, Perciformes, Sparidae)

**DOI:** 10.1186/s13104-016-2058-7

**Published:** 2016-04-29

**Authors:** Seifu Seyoum, Cecilia Puchutulegui, Richard S. McBride

**Affiliations:** Fish and Wildlife Research Institute, Florida Fish and Wildlife Conservation Commission, St. Petersburg, FL USA; Northeast Fisheries Science Center, National Marine Fisheries Service, Woods Hole, MA USA

**Keywords:** Sheepshead, *Archosargus probatocephalus*, Microsatellites, Population structure, Subspecies, Non-specific markers

## Abstract

**Background:**

The sheepshead (*Archosargus probatocephalus*) is found in nearshore waters from Nova Scotia, Canada, to Rio Grande do Sul, Brazil. In the southeastern United States two subspecies are recognized based on a number of meristic characters, primarily counts of melanistic pigment bars. The only previous study based on mtDNA control-region sequence found limited divergence between those subspecies and isolation by distance among 15 locations from Florida (Atlantic Ocean) to Texas (Gulf of Mexico). In the same study, using six sparid microsatellite markers, Bayesian analysis showed that the Gulf and Atlantic sheepshead form a single population. To reinvestigate the fine-scale genetic population structure and examine genetic support for the morphologically classified subspecies, a set of species-specific microsatellite markers was needed.

**Findings:**

Here we report on 24 polymorphic microsatellite markers isolated from sheepshead and screened in 57 specimens from the Indian River, Florida. The average number of alleles per locus was 13.1; mean observed and expected heterozygosities were 0.68 and 0.73, respectively. Nine sparid markers screened for the same specimens showed an average of 8.6 alleles per locus; mean observed and expected heterozygosities were 0.46 and 0.55, respectively.

**Conclusions:**

The polymorphic markers reported here can be used to search for genetic evidence for the morphologically defined subspecies, to elucidate the fine-scale genetic population structure of this broadly distributed coastal species, and to provide an opportunity to directly compare results of population delineation between nonspecific and species-specific markers.

## Findings

The sheepshead (*Archosargus probatocephalus*) is an economically important, estuarine–marine teleost fish that is widely distributed from Nova Scotia, Canada, to Rio Grande do Sul, Brazil [1–3]. They migrate offshore to spawn in late winter and return to estuaries in early spring [3]. Limited movement of sheepshead along the coast could lead to discrete populations among spawning groups, but assuming pelagic eggs disperse freely, this may facilitate gene flow and thwart the formation of population structure [4].

Geographic variation in sheepshead bar counts and growth rates is evident [2, 5]. Variation in melanistic bar patterns initially led to the designation of two subspecies in North America [6], one in the western and northern Gulf of Mexico and another in the eastern Gulf of Mexico and along the Eastern Seaboard. A recent statistical re-evaluation of morphometric data confirmed that these putative subspecies exhibited significantly different numbers not only of melanistic bars but also of several other meristic characters (i.e., scales, gill rakers, and fin rays [2, 7]). This new analyses also indicated the presence of a hybrid zone in the northeastern Gulf [7].

The only study of the genetic population structure of the sheepshead based on six microsatellite loci developed for other sparid showed that the Gulf and Atlantic populations belong to a single panmictic population [7]. In the same study based on mtDNA control region sequence, no specific genetic boundaries were evident that corresponded with the two morphologically defined subspecies mentioned above. In samples from northeast Florida to Texas, mtDNA and microsatellite differentiation was attributed to isolation by distance rather than independent genetic stocks [7]. The mtDNA, however, as a single locus may not reflect enough genealogical histories among populations and because of this has a low-resolution power and often fails to reveal fine-scale population structure. Nonspecific markers may also fall in the category of low resolution molecular techniques, particularly if few are used. Species-specific microsatellite markers, however, may represent genealogical record from the source organism with which to observe population structure in fishes that reside in open, coastal habitats. We postulate that in direct competition species-specific markers should be superior to nonspecific markers and can be helpful in re-examining genetic evidence for the validity of the subspecies and the genetic stock structure of the sheepshead.

Microsatellite loci were isolated following the PIMA (PCR-based isolation of microsatellite arrays) method of Lunt et al. [8], modified by Seyoum et al. [9]. Nuclear DNA (nDNA) was first purified from liver tissue from a single sheepshead via density-gradient ultracentrifugation [10] to minimize competition with mitochondrial DNA during random amplified polymorphic DNA (RAPD) PCRs. RAPD PCRs were conducted in 50-µl reactions containing 15–25 ng of the purified DNA, 50 μM of dNTP mix, 0.25 μl of 0.1–mg/ml BSA; two or three primers randomly chosen from a set of 120, 10-mer RAPD primers (Qiagen Operon Inc.); 5 μl of *Taq* polymerase buffer (10×), 2.5 mM MgCl_2_ (Promega, final concentration); and 1.25 units of Go Taq DNA polymerase (Promega Corporation). The reaction profile was 94 °C for 2 min, 30 × (94 °C for 40 s, 35 °C for 40 s, 72 °C for 45 s), and final extension at 72 °C for 30 min. The purified PCR products (Agilent Technologies) were T-A cloned [11] into plasmid vectors Bluescript PBC KS-Agilent Technologies) that had been tailed with homemade dTTP [12]. About 50 recombinant colonies from each of the PCR products were screened by performing PCR (12.5 μl total reaction volume) containing T3 and T7 vector primers and four repeat-specific primers (5′-[AC]_10_-3′, 5′-[AG]_10_-3′, 5′-[AGC]_5_-3′, 5′-[ACT]_12_-3′). Here, the reaction profile was 94 °C for 2 min, 35 × (94 °C for 30 s, 55 °C for 30 s, 72 °C for 30 s), and final extension 72 °C for 7 min. The PCR products of the clones were run through a 1.5 % low-EEO agarose gel. Colonies that showed two or more bands were further amplified using only the vector primers, the products gel-purified and then cycle-sequenced from both directions using BigDye (version 3.1; Applied Biosystems), and the sequencing products visualized on an Applied Biosystems Prism™ 3130-Avant Genetic Analyzer. Primers were designed for candidate loci using OligoPerfect (Thermo Fisher Scientific); annealing temperature was adjusted to 58–60 °C and fragment size to three categories, 95–115, 125–165, and 185–250, to facilitate multiplex PCR and minimize overlapping of fragment sizes during visualization. Multiplex PCR amplifications for each specimen were carried out in an Eppendorf thermal cycler containing 50–100 ng of total DNA and three optimally selected primers, each forward primer labeled with a unique fluorescent dye. The multiplex PCR reaction consisted of a step-down profile and was as follows: 94 °C for 2 min, 5 × (94 °C for 45 s, 61 °C for 45 s, 72 °C for 45 s); 8 × (94 °C for 40 s, 59 °C for 40 s, 72 °C for 40 s); 10 × (94 °C for 35 s, 57 °C for 35 s, 72 °C for 35 s) 12 × (94 °C for 30 s, 55 °C for 30 s, 72 °C for 30 s) and a final extension at 72 °C for 15 min. The fragments were visualized on an ABI 3130 XL genetic analyzer and genotyped using GeneMapper (version 4.0, Applied Biosystems Inc.). For fragment assays, we used a Gene Scan-500 ROX-labeled size standard.

We extracted total DNA by using a PureGene DNA isolation kit (Gentra Systems Inc., Minneapolis, MN) according to the manufacturer’s instructions from 57 specimens of sheepshead collected from the Indian River, Florida, and used these samples to screen the markers for polymorphisms (Table 1) for a final genetic population structure and analysis of the morphologically defined subspecies.

**Table 1:** Characteristics of 24 polymorphic microsatellite loci in 57 specimens of sheepshead (*Archosargus probatocephalus*) from the Indian River, Florida

Locus	Primer sequence (5′→3′) forward/reverse	Repeat motif	Allele size range	K_a_	*H*_O_	*H*_E_	PIC	GenBank accession no.
Apro01	CACAATCACAAACAATACAAACACAT ACTAATGCCTTTCTGTCTGCA	(AC)_7_/(AC)_24_	119–205	20	0.66	0.85	0.84	KU516013
Apro02	AAAGCGGTTCAGAAGGTTTATTTGT GACAGCACAGAACAGGTTGATATAG	(GT)_17_	100–132	16	0.89	0.89	0.87	KU516014
Apro03	TATAACGATAACACTGCAGAAAGAGC CCCTTTTAAATACGACTAGGACAAAC	(AC)_33_	168–230	24	0.82	0.90	0.88	KU516015
Apro04	GAAATTTCCTTTAACCTTTTCTCTCA GACCAGCTGTTTATAGTTTCAACAAA	(AC)_7_/(AC)_8_	165–205	16	0.83	0.91	0.89	KU516016
Apro05	GAGATTGTTTTGTGCCATCTCTGTT ATATTATTCAGACACACGGGTGGATT	(GT)_22_	149–203	21	0.91	0.90	0.88	KU516017
Apro06	CTAGTCAAAACACCTTTAACCACCTT CCATGTTCGTCTTGAAACACTTTT	(GT)_4_/(GT)_7_	202–224	9	0.85	0.80	0.77	KU516018
Apro10	GCTGATGGTACAGATGACGATAGAG GTAAAACCTTGGAGGTCTCACTCTC	(GT)_6_/(GT)_5_/ (GT)_10_	152–222	17	0.75	0.82	0.79	KU516019
Apro12	TCTACTATCAGCCAAGAGTAAAGCA AGACGTATGTGCATATGTATGGGTA	(CA)_11_	146–162	5	0.70	0.61	0.53	KU516020
Apro13	CTTGACTTCACCTTCACTTCACCTT ATGAACCGAGAAACAACATTACAGT	(AC)_15_/(AC)_15_	152–164	6	0.66	0.70	0.64	KU516021
Apro17	GTATGACTCCAAACCCTCCAGTC CAGCCTTGTGTACTGTTGTTTGTAG	(GT)_7_/(TG)_5_	118–190	21	0.91	0.91	0.89	KU516022
Apro18	AATAAAAGGTGCTGCATGTAGTCATA ATGATACTCCAATCAGTCCACAGTC	(CA)_7_(TA)_4_	167–171	3	0.31	0.29	0.26	KU516023
Apro19	TAACAATGGATTACATCAGTCATGC TTATCCAGCTTAGACTCCCACATAC	(GT)_27_	126–180	23	0.97	0.95	0.94	KU516024
Apro21	CACAGGGATGAGAGTATACAGTACG TTCTTCAAAAAGGCTGTTCTCTTTA	(GT)_9_/(TG)_7_ (CT)_2_(GT)_6_	139–177	10	0.64	0.80	0.76	KU516025
Apro22	AGCTTTGACAGACACTGAGTAACAAC GGGTTCTGATGGATATAGAGTAGCAG	(AC)_16_	141–157	9	0.78	0.80	0.76	KU516026
Apro25	TTCTAAAAATGATGTGTGTATGTCTGT ACCTGGGCCAGAGGATACTACTACT	(TG)_3_/(TG)_8_	122–184	10	0.76	0.81	0.78	KU516027
Apro27	AACAGATTAATGGAATCTCCTTCTGT AAGCACAGCTATTGTTTATGTACGAG	(AC)_8_	163–167	3	0.14	0.14	0.13	KU516028
Apro28	TCTCATACAGTATTTGTCCCTCCTC ATTGCTTAGCATTTAACGAGAAAGA	(TC)_7_TTT(TC)_10_	105–153	9	0.42	0.37	0.35	KU516029
Apro29	GCCTAGCATCATTCTGTCACTCAC ATTCGATGAATTCAGTATGAGTTTGA	(CA)_14_	162–196	11	0.65	0.70	0.67	KU516030
Apro30	CATCCAATTGTGAGAGGTGTCAG ACACGCTAATTGGAGTTTGATGA	(CA)_9_	123–161	17	0.85	0.88	0.87	KU516031
Apro32	CAGAGCTGATATTGCCAGATGAAT TAGTTAACAGTTTGGTGGATTTTCC	(RC)_17_	119–129	4	0.09	0.34*	0.30	KU516032
Apro35	TAAAGAATTCATGGGATAAAGCTCA TTTGTCAGTCTGCATCATGAAGTTA	(CA)_12_	173–219	17	0.86	0.89	0.87	KU516033
Apro38	CTGTGCAGGTCTGTTTGTCTCC CATTAATTCATTGTTAACGGCAAA	(TG)_3_ TC(TG)_5_	117–119	2	0.30	0.49	0.37	KU516034
Apro39	AGTTCCTCTCGTCTCCTTTTCAAG AGAGTGTGTCGTTTTGCATTTCTT	(AC)_14_	126–176	15	0.81	0.84	0.82	KU516035
Apro40	TCAAAATCAATTTAGCAAAATAGGAAA AACGGCTGTAACATTATGTGTGTTT	(G)_13_(A)_5_ (C)_14_/(A)_10_	144–206	27	0.78	0.95*	0.94	KU516036
	Mean			13.1	0.68	0.73	0.70	

*Ka* number of alleles, *H*_*O*_ observed heterozygosity, *H*_*E*_ expected heterozygosity, *PIC* polymorphic information content

* Indicates significant departure from HWE

Microsatellite-marker GENEPOP data was generated using the Microsatellite Marker Toolkit Excel add-on. Genotypic disequilibrium among loci was examined using GENEPOP, version 3.4 [13]. Hardy–Weinberg equilibrium (HWE) expectation, observed and unbiased expected heterozygosity estimates with Bonferroni correction, number of alleles, and polymorphic information content, a value that is indicative of a measure of the informativeness of a genetic marker for linkage studies were estimated using the program CERVUS, version 3.0.7; [14].

There were significant departures from HWE after Bonferroni correction at two loci and no linkage disequilibrium at any pair of loci. Analyses using the program CERVUS suggested that the observed nonconformance to HWE may have resulted from the presence of null alleles at those loci. The average number of alleles per locus was 13.1 (range 2–27); the mean observed and expected heterozygosities were, respectively, 0.68 (range 0.09–0.97) and 0.73 (range 0.29–0.95). The relative informativeness of each marker ranged from 0.24 to 0.93 (mean 0.70), with 11 of the 24 loci between 0.82 and 0.94.

To build the basis for a preliminary comparative analysis between nonspecific and species-specific markers, 9 sparid markers were characterized for the 57 specimens (Table 2; 4 specimens had incomplete genotypes and were excised). The result showed that the sparid markers provided 8.6 alleles per locus, but much lower means of observed and expected heterozygosities, 0.46 and 0.55, respectively. Overall, the species-specific markers show greater variability and would be of a higher resolution power than the 9 sparid microsatellite DNA loci.

**Table 2:** Characteristics of 9 adopted sparid microsatellite DNA loci in 53 specimens of sheepshead (*Archosargus probatocephalus*) from the Indian River, Florida

Source organism	Primer sequence (5′→3′) forward/reverse	Repeat motif	Allele size range	K_a_	*H*_O_	*H*_E_	PIC	GenBank accession no.	Ref
*Pagrus auratus*	GTCCGACTCCACTCCATTCCTCT GTGCTCGATCCCTTGTGCTGATA	(CTGT)_7_	124–126	12	0.60	0.63	0.59	AY696589	[18]
*Acanthopagrus butcheri*	GGTGCGTGCATTGTTAATGTGT GATCTGCTTTCCTTTGACTCAGC	(TG)_24_	90–126	15	0.87	0.89	0.87	AF284352	[19]
*Acanthopagrus schlegelii*	AGGCATTTCCGCACACTAAC CAAACAAGAGCCTGGAGGAG	(GT)_12_	198–218	8	0.64	0.63	0.55	AB095014	^a^
*Diplodus vulgaris*	GCCGGGCTCGACATTGACACTGAA GCAGCCAGCAGAGCTTAAAGAACT	(CA)_11_	260–266	2	0.04	0.04	0.04	EF064291	[20]
*Diplodus vulgaris*	GCGGTTATGTATACGTTGCGTTTA TTGGCGTTGAACAGAAGTCAGACA	(CA)_13_	238–248	3	0.15	0.18	0.17	EF064292	[20]
*Pagrus auratus*	AATCTTGACAGCGCCCTTTA GCAGCGCACAGATAAACAAA	(GT)_16_	156–204	18	0.92	0.89	0.87	AF202881	[21]
*Pagrus auratus*	GGACAGAGAGGGAGTGGATG GCTCCTCTGCCTGTATCTGG	(AG)_16_	216–248	13	0.72	0.92	0.90	AF202885	[21]
*Paqrus major*	TTCCAATGTGCCTTTATGC CAAATTCCCAAGGTCATCC	(GT)_24_	120–128	3	0.11	0.39	0.32	AB042989	[22]
*Pagrus auratus*	GTGCGTGTGTGTGTGTTGG^a^ ATCCTCCTCCACTCCATG	(TG)_21_	160–170	3	0.07	0.38	0.31	AB042989	[22]
	Mean			8.6	0.46	0.55	0.51		

Four specimens did not amplify for three or more loci and were excised

*Ka* number of alleles, *H*_*O*_ observed heterozygosity, *H*_*E*_ expected heterozygosity, *PIC* polymorphic information content, *Ref* references

^a^ Direct submission-Jeong et al. (NCBI) 2007

Costs of developing species-specific markers remain a concern. Compared with the enrichment protocol of developing microsatellite markers, the PIMA method requires less expertise, less time, and, so, less expense. The method’s drawback is that it results mostly in dinucleotides, which are more plentiful because their mutation rate is at least six times that of other short tandem repeats (STR). Although tri- and tetranucleotides may not be more variable than dinucleotide, they’re definitely easier to score and lead to a lower genotyping error. The various kinds of tri- tetra- penta- and hexanucleotides can be obtained with the PIMA method, but with specific designs and much more laborious search.

Nonspecific markers are used to circumvent the expertise and the expense required to develop specific markers, but they may not have adequate resolution power to reveal fine-scale population structure. In a direct comparative study, the use of 11 highly polymorphic red drum (nonspecific) markers failed to delineate the eastern and western Gulf spotted seatrout (*Cynoscion nebulosus*) samples as belonging to different clusters compared to only three spotted seatrout markers that accomplished the task (Seyoum et al. in preparation). While nonspecific microsatellite loci are widely used [15, 16] and could reveal strong genetic breaks between populations, there has been no study to directly compare specific vs. nonspecific microsatellite loci on equal terms of allelic variability in revealing fine genetic breaks between populations. The inadequate (and possibly misleading) results of the previous work on the study of the genetic population structure of the sheepshead that used non-specific markers may be offset by these new, species-specific markers developed in this study. We believe that these suite of species-specific markers will shed light on the stock structure of the sheepshead and on the validity of the morphologically classified subspecies. The stock structure of this fish ought to be determined now, especially because as attention increases toward regulation of other Gulf species, the sheepshead is now the target of greater demand and pressure of in the Gulf of Mexico [17]. Updated knowledge of sheepshead stock structure will help direct relevant management actions to protect this species at the appropriate spatial scale across its range.
